# Birt–Hogg–Dubé syndrome in an Indonesian patient with folliculin gene mutation

**DOI:** 10.1002/rcr2.199

**Published:** 2016-10-27

**Authors:** Wiwien Heru Wiyono, Fariz Nurwidya, Hario Baskoro, Andika Chandra Putra

**Affiliations:** ^1^ Department of Pulmonology and Respiratory MedicineFaculty of Medicine, Universitas IndonesiaJakartaIndonesia

**Keywords:** Birt–Hogg–Dubé syndrome, FLCN gene mutation, multiple lung cysts

## Abstract

Birt–Hogg–Dubé (BHD) syndrome is a rare autosomal dominant disorder that affects the skin, kidney, and lungs. Affected individuals have an increased risk of developing multiple cysts in the lungs and a spontaneous pneumothorax. Germline mutations in the folliculin (FLCN) gene have been confirmed as the aetiology of BHD syndrome. A 51‐year‐old Indonesian female presented with recurrent spontaneous pneumothorax, multiple cysts in both lungs, and a renal cyst on magnetic resonance imaging (MRI). Blood sampling was performed to extract genomic DNA from peripheral blood leucocytes. We identified an aberrant band in the DNA fragment derived from FLCN exon 6. Moreover, direct sequencing of FLCN exon 6 by denaturing high‐performance liquid chromatography (DHPLC) showed a pathogenic mutation, which caused premature termination of folliculin protein translation. This is the first reported case of BHD syndrome in an Indonesian patient confirmed by detection of a FLCN exon 6 mutation.

## Introduction

Birt–Hogg–Dubé syndrome (BHDS) is a rare hereditary disease that presents with multiple pulmonary cysts, fibrofolliculomas, and recurrent pneumothorax. It was first described in 1977 as a familial dermatologic disorder by Birt and colleagues 1. The prevalence of BHDS is difficult to determine because this disease is very rare. BHDS is predicted to be responsible for 5–10% of patients with primary spontaneous pneumothorax 2. BHDS is caused by germline, predominantly truncating mutations in the folliculin (*FLCN*) gene located on chromosome 17, which encodes a highly conserved tumour suppressor protein folliculin 2. *FLCN* gene mutations were first discovered by Nickerson and colleagues in 2002 and have provided the basis for our understanding of the role of *FLCN* in pathways common to skin, lung, and kidney development 2. In Asia, BHDS has been identified in several countries; however, there have been no reports of BHDS in a Southeast Asian patient.

## Case Report

**Figure 1:**
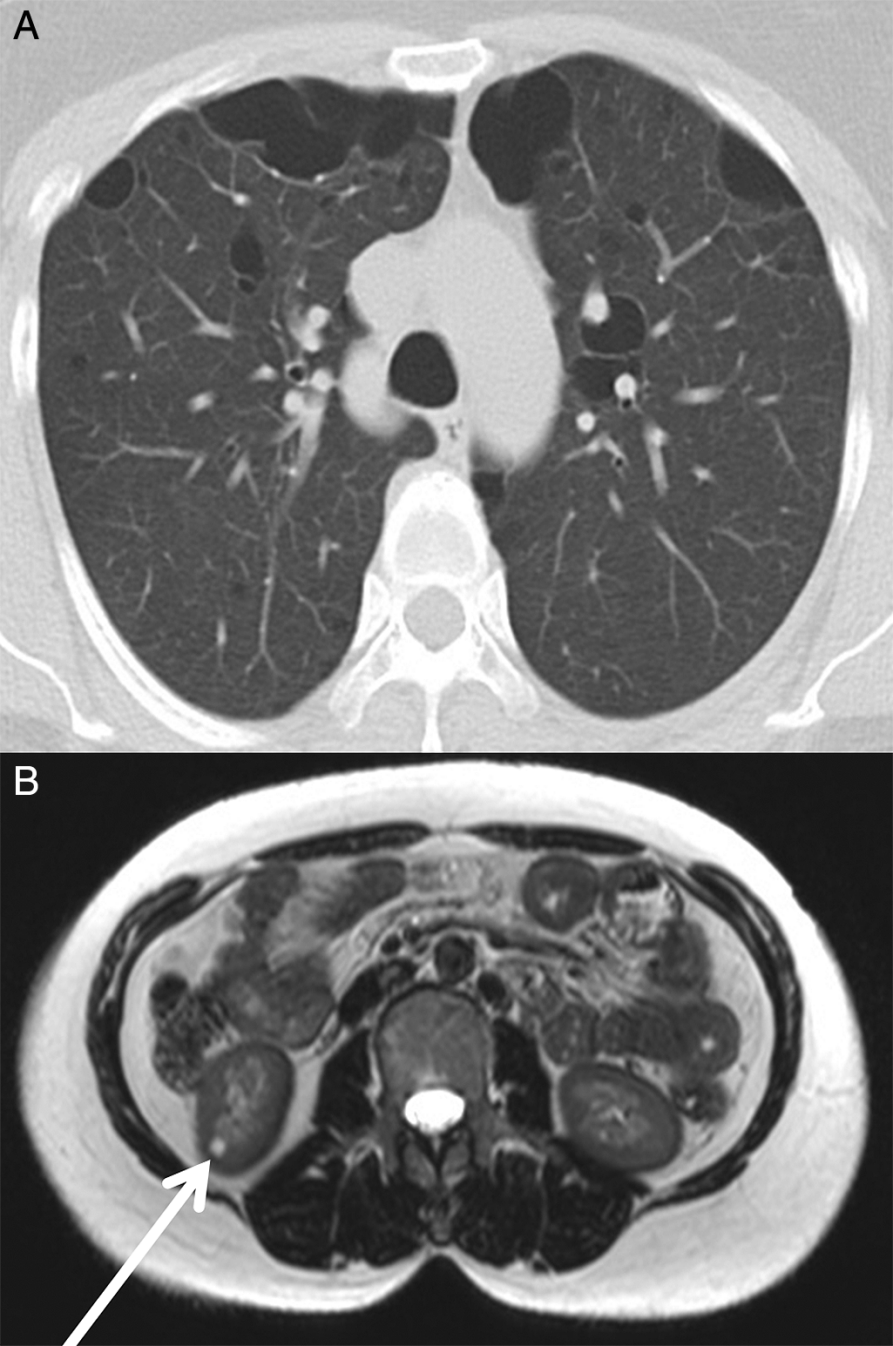
(A) Thoracic computed tomography of the patient showed multiple bilateral pulmonary cysts. (B) Abdominal magnetic resonance imaging revealed a renal cyst (white arrow).

**Figure 2:**
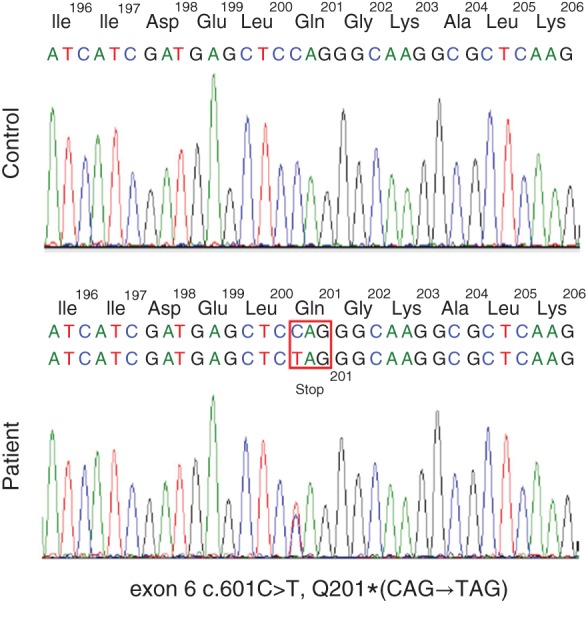
Sequence chromatogram of the FLCN gene showed a mutationin exon 6.

A 51‐year‐old Indonesian female, with a medical history of multiple lung cysts, presented to our hospital complaining of shortness of breath but without a cough, chest pain, or fever. A computed tomography (CT) scan of her chest demonstrated multiple cysts on both lungs as previously noted (Fig. 1A). Her family history revealed that her mother had a history of renal tumour, although the patient was not sure whether it was malignant or not. We performed magnetic resonance imaging (MRI) of her abdomen and a renal cyst was noted (Fig. 1B). The combination of pulmonary and renal cysts raised the possibility of BHDS. This clinical suspicion was confirmed on folliculin (*FLCN*) gene mutation analysis that was carried out on DNA isolated from peripheral blood leucocytes. Each exon of the *FLCN* gene was amplified by polymerase chain reaction (PCR) and subjected to denaturing high‐performance liquid chromatography (DHPLC). This detected an aberrant band in the DNA fragment derived from exon 6. Direct sequencing identified a pathogenic mutation c.601C > T (see sequence chromatogram; Fig. 2). This nonsense mutation triggers a premature stop codon at Q201 and early termination of protein translation. Family members of the patient declined screening.

## Discussion

This was a rare case of multiple cysts in the lung accompanied by kidney cyst in a patient with a maternal history of a renal tumour. Confirmation of BHDS was provided by *FLCN* gene mutation analysis. The availability of DNA‐based diagnosis has allowed insight into the great variation in clinical manifestation of BHDS. For example, some patients with a specific mutation in the *FLCN* gene may have a higher risk of recurrent spontaneous pneumothorax 3, although all BHDS patients have a heightened risk. In addition to cutaneous fibrofolliculomas, pulmonary cysts and repeated episodes of pneumothorax are the key diagnostic features of BHDS. About 80% of BHDS patients have multiple pulmonary cysts on their CT scans. These are typically small thin‐walled cysts, are adjacent to the fissures, often involve the lower lobes, are prone to rupture, and consequently a pneumothorax may be the initial presentation of this disease.

Multiple biopsy‐proven fibrofolliculomas are diagnostic for BHDS and occur in >80% of BHDS‐affected individuals. However, Tomassetti et al. 3 reported BHDS‐affected patients without skin papules, and fewer than 50% of a cohort of Japanese Asian FLCN mutation carriers presented with skin papules, suggesting that lung and kidney lesions may be more informative than fibrofolliculomas as the diagnostic criteria for BHDS in the Japanese Asian population 4.

Renal tumours, most commonly chromophobe or hybrid oncocytic renal tumours, may develop in up to one‐third of BHDS‐affected individuals 5. Of note, our patient reported a maternal family history of renal tumour, although an abdominal MRI revealed only a single renal cyst in our patient, which has been infrequently reported in BHDS. It is unknown whether this benign renal cyst will progress to a renal tumour in the future.

In accordance with recently published findings, our results help to raise further awareness about BHDS 2. To the best of our knowledge, this report presents the first published case of BHDS in a patient from Southeast Asia that was confirmed by a *FLCN* exon 6 nonsense mutation predicted to truncate the folliculin protein. Given that this patient presented only with pulmonary manifestations without renal tumour or skin papules, this case report should raise awareness among pulmonologists in Southeast Asia to consider genetic testing for BHDS in a patient who presents with multiple lung cysts with or without history of pneumothorax, especially when skin papules and/or renal tumours are also present in the patient or his/her family history. Conversely, BHDS may be suspected in a patient who presents with bilateral multifocal renal tumours with chromophobe or hybrid oncocytic histology, and screening for pulmonary or cutaneous manifestations may be recommended. Owing to the increased risk of renal cancer in BHDS patients, individuals with a pathogenic *FLCN* mutation should undergo periodic screening by abdominal MRI. BHDS patients presenting with lung cysts should be made aware of the risk of developing a spontaneous pneumothorax and instructed to avoid activities that would suddenly change intra‐thoracic pressure (i.e., scuba diving). Pleurodesis may be considered in BHDS patients with previous spontaneous pneumothorax in order to avoid recurrence.

### Disclosure Statements

No conflict of interest declared.

Appropriate written informed consent was obtained for publication of this case report and accompanying images.
